# Titanium Dioxide Nanoparticles As Guardian against Environmental Carcinogen Benzo[alpha]Pyrene

**DOI:** 10.1371/journal.pone.0107068

**Published:** 2014-09-12

**Authors:** Anupam Dhasmana, Qazi Mohd. Sajid Jamal, Snober Shabnam Mir, Madan Lal Bramha Bhatt, Qamar Rahman, Richa Gupta, Mohd. Haris Siddiqui, Mohtashim Lohani

**Affiliations:** 1 Environmental Carcinogenesis and Toxicoinformatics Laboratory, Department of Bioengineering & Biosciences, Integral University, Lucknow, Uttar Pradesh, India; 2 Department of Radiation Oncology, Dr. Ram Manohar Lohia Institute of Medical Sciences, Lucknow, Uttar Pradesh, India; 3 Science & Technology, Amity University Campus, Malhaur, Gomti Nagar, Lucknow, Uttar Pradesh, India; 4 Developmental Toxicology Division, Indian Institute of Toxicology Research, CSIR, Qaiserbagh, Lucknow, Uttar Pradesh, India; Indian Institute of Toxicology Research, India

## Abstract

Polycyclic aromatic hydrocarbons (PAH), like Benzo[alpha]Pyrene (BaP) are known to cause a number of toxic manifestations including lung cancer. As Titanium dioxide Nanoparticles (TiO_2_ NPs) have recently been shown to adsorb a number of PAHs from soil and water, we investigated whether TiO_2_ NPs could provide protection against the BaP induced toxicity in biological system. A549 cells when co-exposed with BaP (25 µM, 50 µM and 75 µM) along with 0.1 µg/ml,0.5 µg/ml and 1 µg/ml of TiO_2_ NPs, showed significant reduction in the toxic effects of BaP, as measured by Micronucleus Assay, MTT Assay and ROS Assay. In order to explore the mechanism of protection by TiO_2_ NP against BaP, we performed *in silico* studies. BaP and other PAHs are known to enter the cell via aromatic hydrocarbon receptor (AHR). TiO_2_ NP showed a much higher docking score with AHR (12074) as compared to the docking score of BaP with AHR (4600). This indicates a preferential binding of TiO_2_ NP with the AHR, in case if both the TiO_2_ NP and BaP are present. Further, we have done the docking of BaP with the TiO_2_ NP bound AHR-complex (score 4710), and observed that BaP showed strong adsorption on TiO_2_ NP itself, and not at its original binding site (at AHR). TiO_2_ NPs thereby prevent the entry of BaP in to the cell via AHR and hence protect cells against the deleterious effects induced by BaP.

## Introduction

Human exposure to xenobiotics is almost inevitable. Most of the human cancers are caused due to exposure to xenobiotics including PAHs and hence they are ultimately preventable. PAHs are produced during the combustion processes of organic materials during industrial and other human activities, like processing of coal and crude oil, vehicle traffic and cigarette smoke. PAHs may cause carcinogenesis by damaging the DNA and/or a number of proteins [1]. The benzo[a]pyrene (BaP) is one of the most common PAHs and is a byproduct of grilled foods, tobacco, cigarette smoke and fuel combustion. BaP has long been correlated to a range of human cancers, predominantly lung and skin cancer [2], [3]. The carcinogenic properties of BaP in particular are mostly explained by their capability to induce DNA damage. BaP is the only PAH listed in group 1 by the International agency for research on cancer [4], and has thus been broadly considered and constitutes the reference compound for assessing toxicity of exposure to mixtures in the toxic equivalent factors approach [5].

BaP enters the cell via aryl hydrocarbon receptor (AHR) [6], along with stimulating the AHR to activate transcriptional regulation of xenobiotic response element (XRE) and genes coding for xenobiotic metabolizing enzymes such as cytochrome P450s (CYPs), UDP glucuronosyltransferase UGT1A6, NAD(P)H:quinone oxidoreductase-1 (NQO1), aldehyde dehydrogenase (ALDH3A1), and various glutathione-S-transferases [7].

After the enzymatic metabolism, BaP is converted to benzo[a]pyrene-7,8-diol-9,10-epoxides (BPDE), a crucial carcinogenic metabolite of BaP, that reacts primarily with the N2 position of guanine residues and to a minor coverage with the N6 position of adenine residues in DNA [8] to form bulky adducts that block DNA synthesis by replicative or high fidelity DNA polymerases [3].

Recently, titanium dioxide nanoparticles have been employed in scavenging the high molecular weight polycyclic aromatic hydrocarbons (PAHs) from the contaminated soils [9]. The scavenging capacities of the nanoparticles for PAH and other toxicants could be attributed to their higher affinity towards the xenobiotics due to surface chemistry, large surface area and other intrinsic properties of nanoparticles. Some studies also have shown that titanate nanotube has the capacity to scavenge the PAHs from water sample from the environment [10]. The nanoform of TiO_2_ for example titanate nanoSheets (TNS) and titanate nano tubes (TNT) have also been synthesized and used as additives for removing harmful compounds from cigarette smoke [11] including nicotine, tar, ammonia, hydrogen cyanide, selected carbonyls and phenolic compounds. Interestingly, TNT exhibits highly efficient reduction capability for most of the harmful compounds. This might be related to the intrinsic properties of TNT [11]. TiO_2_ is a naturally occurring oxide of http://www.absoluteastronomy.com/topics/Titanium titanium, and is biologically inert at lower doses, whereas, at higher doses it may induce slight toxicity and even apoptosis [12].

Considering this, we designed the present study in order to explore whether the discussed property of the TiO_2_ NPs could be exploited in the biological system to safeguard against the deleterious effects of PAHs exposure. We also explored the doses of TiO_2_ NPs, at which they provide maximum protection. Further, *in silico* experiments were also performed using bioinformatics tools, to attain insight of mechanism of protection.

## Materials and Methods

### 1. Reagents and consumables

Most of the specified chemicals, reagents, diagnostic kits etc were purchased from Sigma Chemical Company Pvt. Ltd. (St. Louis, MO, USA). Cell culture media, PBS, antibiotic-antimycotic were purchased from Hi-Media (Hi-Media Pvt. Ltd., Mumbai, India).

#### 1.1 Titanium dioxide Nanoparticles

Anatase form of TiO_2_ NPs (d<25 nm, specific surface area 200–220 m^2^/g) without any coating were purchased from Sigma Aldrich (St. Louis, Missouri, USA, Cat no. 637254). Particles were sterilized by heating to 120°C for 2 h and suspended in phosphate-buffered saline (stock: in 1 µg/ µl PBS). The mean hydrodynamic diameter and zeta potential (*ζ*) of the TiO2 NPs suspension in complete medium as determined by dynamic light scattering (DLS) measurement was 434.1 nm and −7.83 mV, respectively, as described previously by us [13].

#### 1.2 Cell culture and treatment conditions

A549 cells (lung carcinoma cells) were obtained from the cell bank of NCCS Pune, Maharashtra, India, and were grown in a humidified atmosphere with 5% CO2 at 37°C. The cells were cultured in Dulbecco's Modified Eagle's Medium, supplemented with 10% fetal bovine serum and 1% antibiotic and anti-mycotic solution, according to the standard procedure. Prior to use in the experiments, cell viability was estimated using trypan blue dye exclusion assay following the protocol as described earlier [14] and batches showing viability more than 95% were used for further experiments.

### 2. Dose optimization

Different doses of BaP (10 µM, 25 µM, 50 µM and 75 µM) were tested in A-549 cells for the selection of most suitable doses for various assays in our study.

#### 2.1 Micronucleus Assay

Three different sets of cells were treated with: i) different doses of TiO_2_ NPs (0.1, 0.5 and1.0 µg/ml), ii) highest genotoxic dose i.e. 25 µM of BaP, and iii) co-exposure of BaP (25 µM) and different doses of TiO_2_ NPs (0.1 µg/ml, 0.5 µg/ml, and 1.0 µg/ml).

#### 2.2 MTT assay

Three different sets of cells for each of three different time periods (6, 12 and 24h) were treated with: i) different doses of TiO_2_ NPs (0.1, 0.5 and1.0 µg/ml), ii) highest cytotoxic dose i.e. 75 µM of BaP, and iii) co-exposure of BaP (75 µM) and different doses of TiO_2_ NPs (0.1 µg/ml, 0.5 µg/ml, and 1.0 µg/ml).

#### 2.3 ROS Assay

Three different sets of cells for each of four different time periods (2, 6, 12 and 24h) were treated with: i) different doses of TiO_2_ NPs (0.1, 0.5 and1.0 µg/ml), ii) highest ROS producing dose i.e. 50 µM of BaP, and iii) co-exposure of BaP (50 µM) and different doses of TiO_2_ NPs (0.1 µg/ml, 0.5 µg/ml, and 1.0 µg/ml).

### 3. Methodology

#### 3.1 Micronucleus Assay

The genetic damage was assessed by MN assay as described earlier [15]. In brief, A549 cells were grown on cover slips for 24 h in 6 well plates. The cells were exposed to different treatment conditions as discussed and incubated for 24h. The cells were fixed in cold fixative and stored at −20°C for at least 30 min. DNA staining was performed using bisbenzimide (1 µg/ml; Hoechst 33258; Sigma, St. Louis, MO, USA) for 4 min. the cells were washed in phosphate buffered saline (PBS), and were mounted on slide for microscopy. 5000 cells were analyzed for each condition and results were expressed as MN/1000 cells. MN smaller than one-third the diameter of the nucleus were scored under a fluorescent microscope at 630× magnification.

#### 3.2 MTT Assay

Percentage cell viability was assessed using the 3-(4, 5-dimethylthiazol-2-yl)-2, 5-diphenyl tetrazolium bromide (MTT) assay as described earlier [16]. In brief, the cells (1×10^4^) were allowed to adhere for 24 h in 5% CO2 at 37°C and 20% humidity in 96-well culture plates. After the exposure for 6h, 12h and 24h, MTT (5 mg/ml of stock in PBS) was added (10 µl/well in 100 µl of cell suspension), and plates were incubated for another 4h. At the end of incubation period, the reaction mixture was carefully taken out and 200 µl of dimethyl sulfoxide was added to each well, the contents were mixed well by pippeting up and down several times. The plates were kept on rocker shaker for 10 min at room temperature and then read at 550 nm using multiwell microplate Reader (Multi Skan, Thermo Scientific). Untreated sets were run under identical conditions and served as basal control.

#### 3.3 ROS Assay

ROS generation was assessed in A549 cells using 2',7'-diclorodihydrofluorescein di-acetate (DCFH-DA, Sigma Aldrich, Missouri, USA) dye as fluorescence agent. ROS generation was performed as described earlier [12]. The cells (1×10^4^ per well) were seeded in 96-well black bottom culture plates and allowed to adhere them for 24h in CO_2_ incubator at 37°C. The medium was then aspirated and cells were exposed to different conditions as describes for 2h, 6h, 12h and 24h. On the completion of respective exposure periods, cells were incubated with DCFH-DA (10 mM) for 30 min at 37°C. The reaction mixture was then aspirated and replaced by 200 ml of PBS in each well. The plates were kept on rocker shaker for 10 min at room temperature in the dark. Fluorescence intensity was measured using multiwell microplate reader (Multi Skan, Thermo Scientific) on excitation wavelength at 485 nm and emission wavelength at 528 nm. The data were expressed as percentage of the unexposed control.

### 4. *In silico* study

#### 4.1 Preparation and Validation of AHR

**Figure 1:**
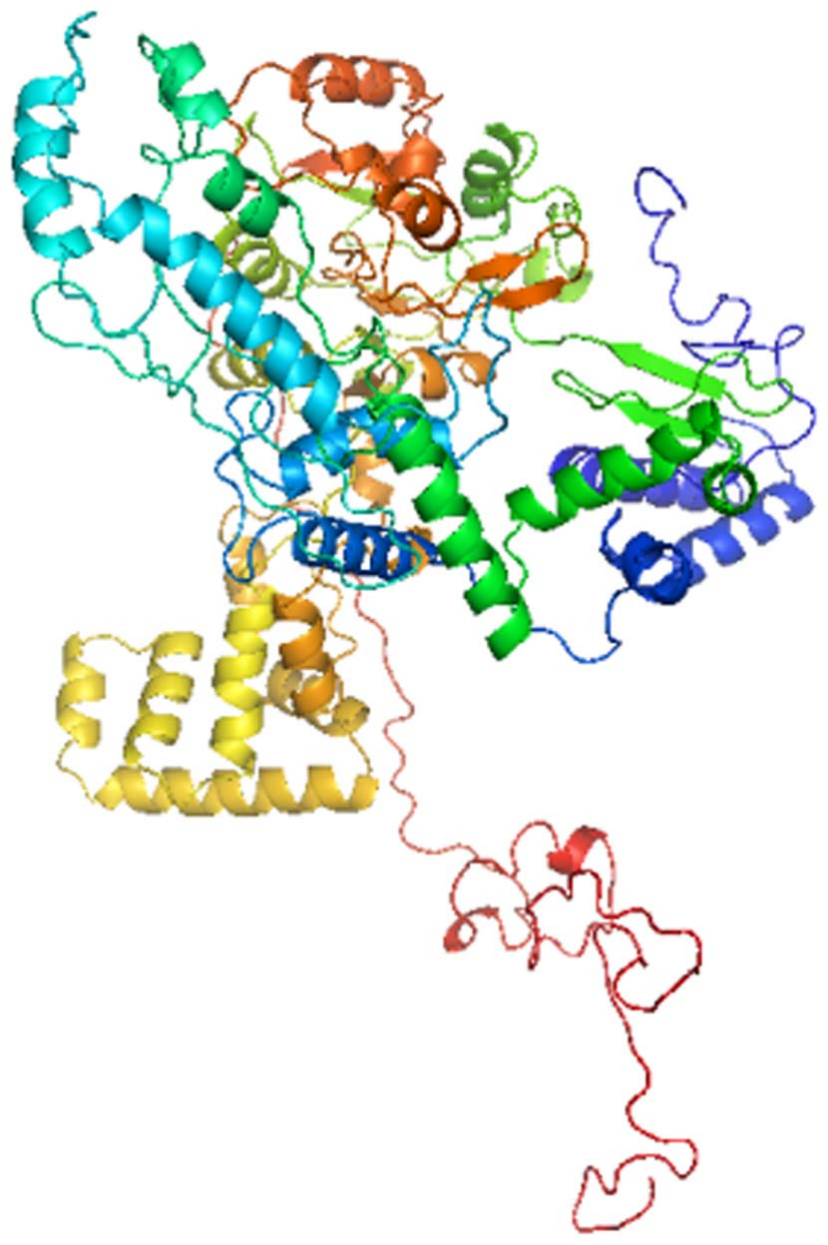
AHR structure as predicted with help of I-TASSER online server.

PDB structure of aryl hydrocarbon receptor (AHR Uniprot entry - P35869) is not available in the PDB databank. I-TASSER [17] online server was utilized for the *ab-initio* modeling to build the 3D structure of AHR as shown in Figure 1, and validated by the approach of Ramacharndran Plot by using RAMPAGE [18].

AHR 3D structure has been submitted in Protein Model Data Base, (PMDB ID- PM0078981) [19], a Protein Data Base which collects three dimensional protein models obtained by structure prediction methods.

#### 4.2 Preparation of Benzo[a]Pyrene

**Figure 2:**
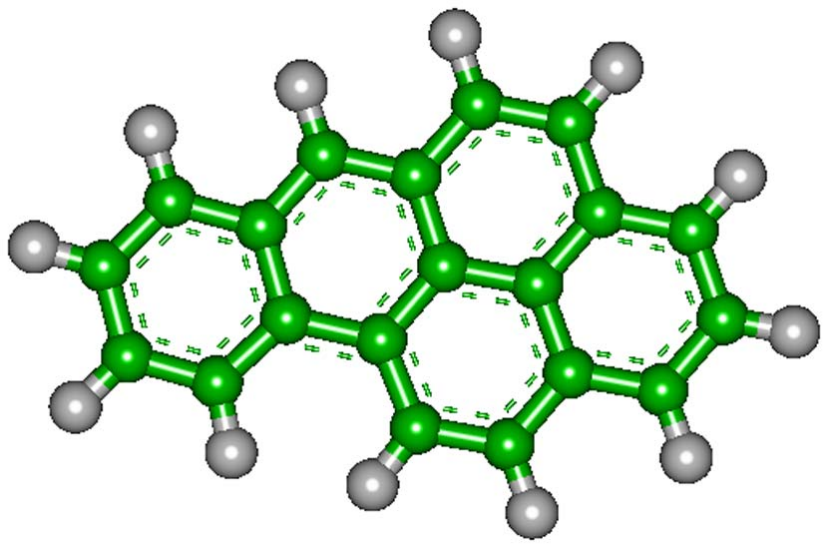
BaP structure as genrated with help of CORINA online server.

The SMILES (Simplified Molecular Input Line Entry Specification) notations of the BaP were obtained from the ZINC database (ZINC ID - 01530818). The 3D-structure of BaP was generated *de novo*, via the internet (http://molecular-networks.com/products/corina), by program CORINA on server running in Computer-Chemie-Centrum, Universiy of Erlangen-Nurnburg, Germany. It is a rule- and data-base system, that automatically generates 3D atomic coordinates from the constitution of a molecule as expressed by connection table or linear code as shown in Figure 2.

#### 4.3 Construction of Anatase TiO_2_ Nanoparticle

After studying the anatase crystal structure, we found that anatase is the thermodynamically favored phase. According to the anatase lattice parameters, the tetragonal crystal have lengths (A, B and C) as; A = B = 3.782 Å, C = 9.502. Å and angles (alpha, beta and gamma) as; Alpha  =  beta  =  gamma  = 90^o^ [20].

The Accelrys Discovery studio 2.5 program was found to be the most suitable software for the designing of TiO_2_ anatase crystal structure.

**Figure 3:**
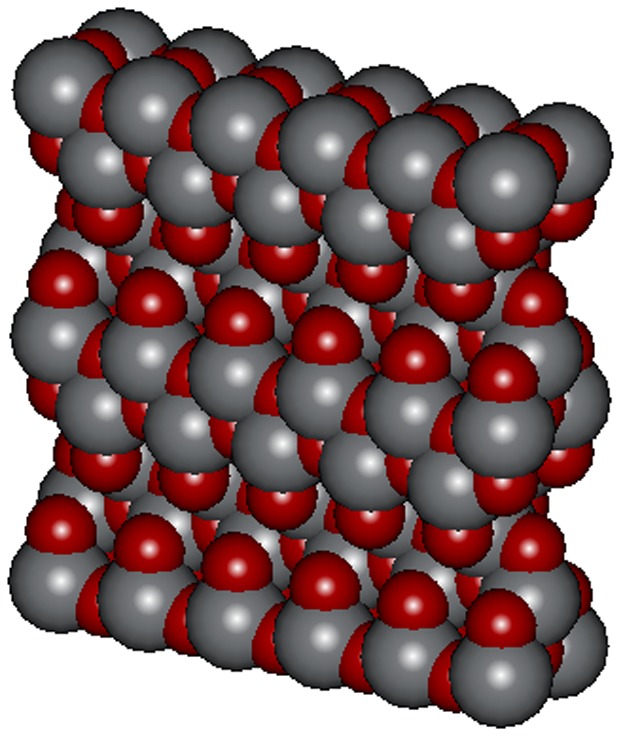
TiO_2_ NP structure as constructed with help of DISCOVERY STUDIO 2.5.

After the construction of Unit cell of TiO_2_ anatase by using the anatase lattice parameters, a surface was created and this unit cell was extended in the desired directions (axis) creating a new surface of TiO_2_ comprising [1,0,1] of 5 unit cells in × direction and 2 unit cells in the Z direction. This gave a surface of dimensions 1.891×0.3782×1.9004 nm^3^ as shown in Figure 3.

#### 4.4 Docking Study

All the *in silico* docking analyses were performed using PatchDock [21]. The AHR was docked with the BaP, as well as TiO_2_ NP. The resultant pdb file obtained after AHR and TiO_2_ NP docking was used as AHR-TiO_2_ NP complex, and was docked with BaP by uploading the receptor and molecules in PatchDock Server, an automatic server for molecular docking. Clustering RMSD was chosen as 4.0 Å.

### 5. Statistical analysis

All the experiments were performed in triplicates and were repeated twice. The final results were expressed as mean of the values obtained from all experiments. The standard error of mean (SEM) was also calculated. Statistical analysis was performed by one-way analysis of variance (ANOVA) using Newman- Keuls test to compare all the groups by graph pad prism3. In all the cases, p<0.05 was considered as significant.

## Results

### 1. Dose optimization

Different doses of BaP (10 µM, 25 µM, 50 µM and 75 µM) were tested in A-549 cells to determine the most suitable dose for each assay in the study.

**Figure 4:**
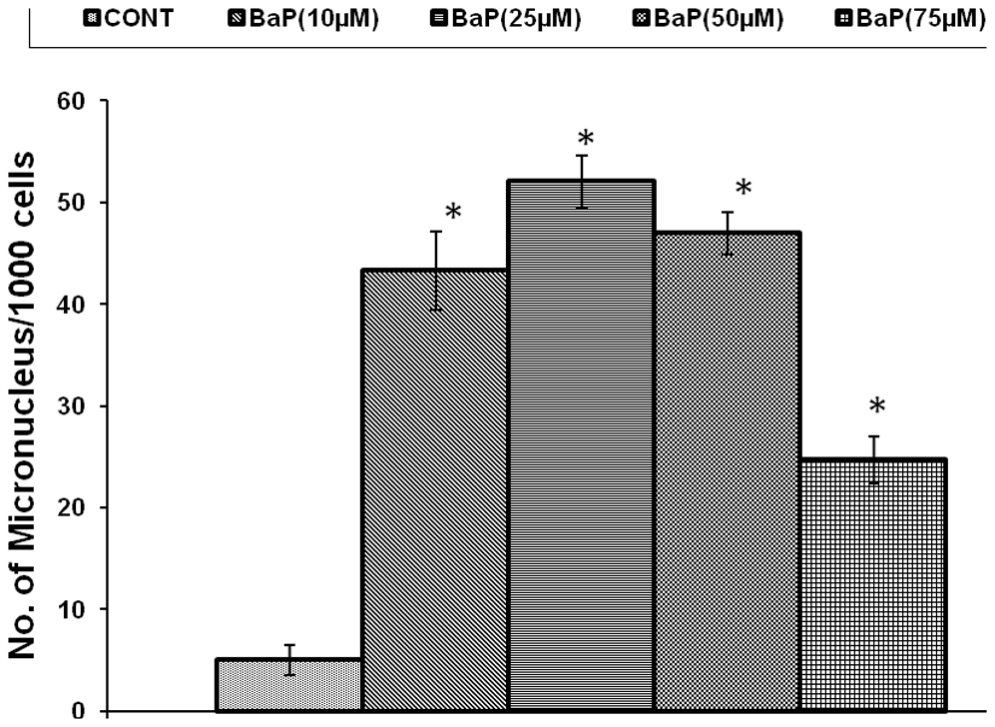
Number of micronucleus/1000 cells, induced by various concentrations of BaP after 24 h exposure, *p<0.05 considered as significant. BaP: Benzo[alpha]Pyrene; MN: Micronucleus.

The MN induced were 43.33±3.844, 52±2.646, 47±2.082 & 24.67±2.333 MN/1000 cells at 10 µM, 25 µM, 50 µM and 75 µM BaP concentrations respectively after 24h exposure, against 5±1.528 MN/1000 in control (unexposed) cells as shown in Figure 4. 25 µM concentration was selected for MN assay in further experimentation as highest genotoxicity was observed at this dose, above this concentration BaP became cytotoxic.

**Figure 5:**
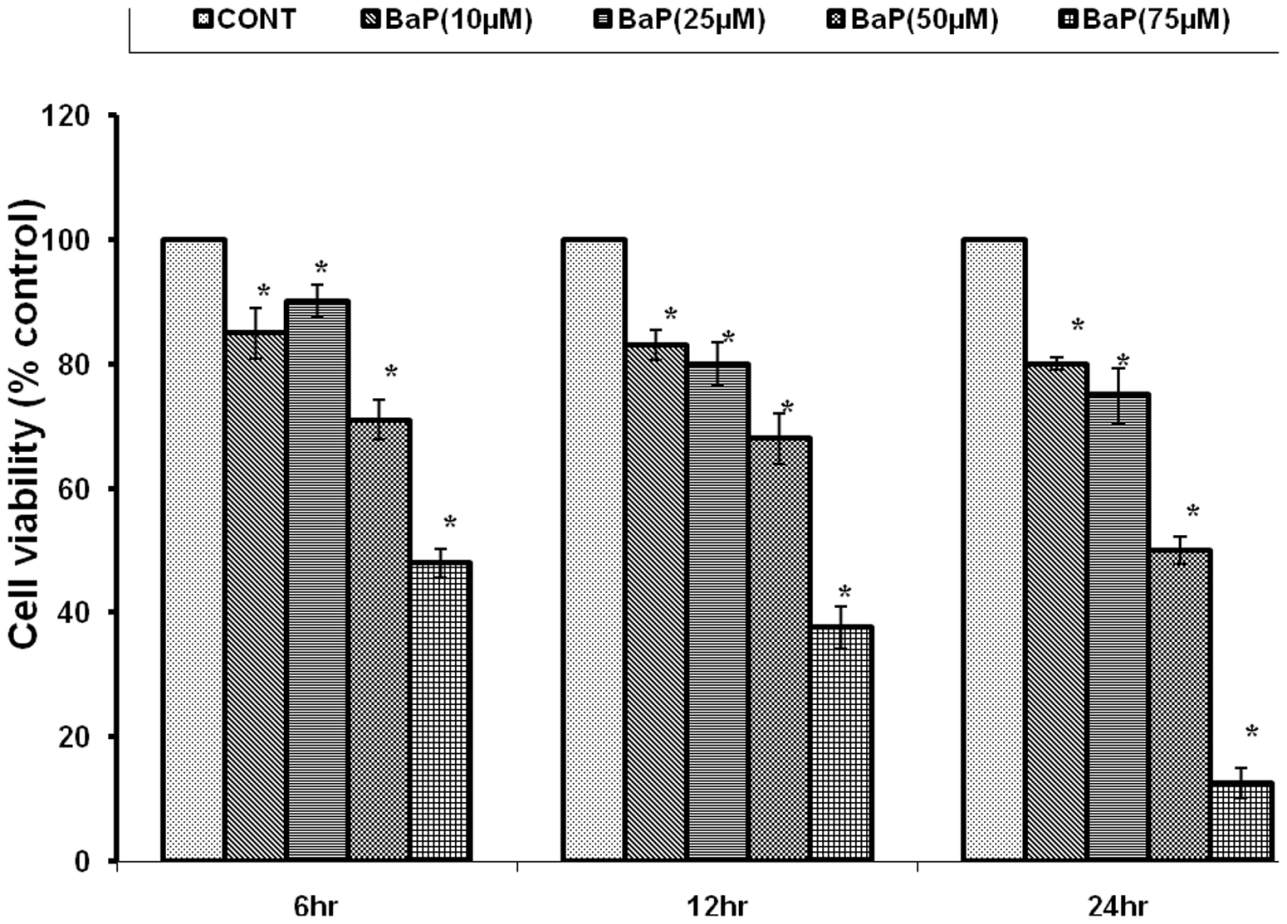
Identification of cell viability after 6, 12 & 24 h of exposure to various concentrations of BaP. *p<0.05 indicates significance. MTT: 3-(4, 5-dimethylthiazol-2-yl)-2, 5-diphenyl tetrazolium bromide; BaP: Benzo[alpha]Pyrene.

The cell viability, following exposure to 10 µM, 25 µM, 50 µM and 75 µM of BaP concentrations was observed as 85±4.041, 90±2.646, 71±3.215, and 48±2.333% after 6h, 83±2.43, 80±3.44, 68±4.152, and 37.68±3.423% after 12h exposure, and 80±1.01, 75±4.381, 50.2±2.153, and 12.72±2.412% after 24h exposure respectively, against control (unexposed) cells, as shown in Figure 5. 75 µM concentration was selected for MTT assay in further experimentation as highest cytotoxicity was observed at this dose.

**Figure 6:**
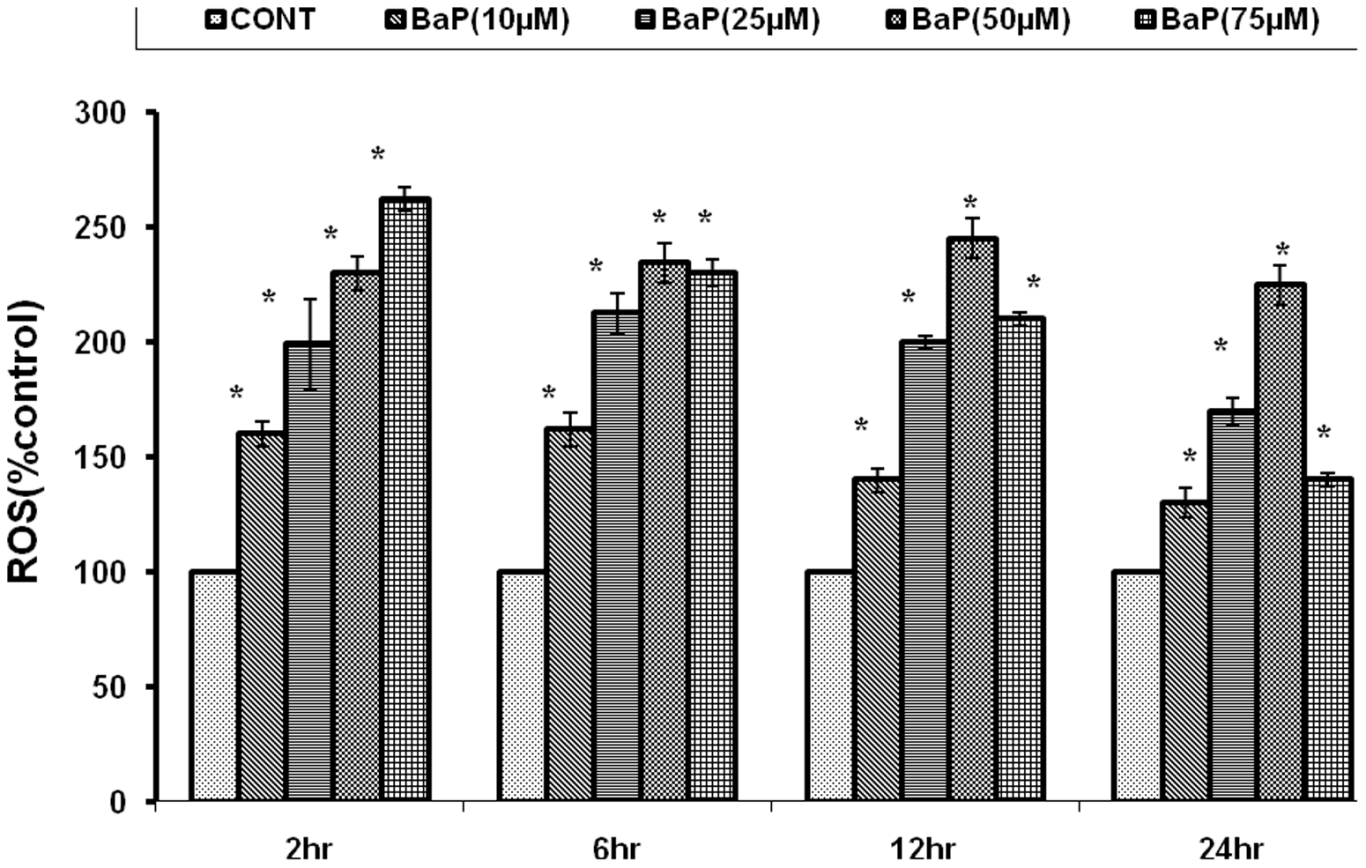
Percentage change in ROS generation following 2, 6, 12 and 24 h of exposure to various concentrations of BaP. *p<0.05 considered as significant. BaP: Benzo[alpha]Pyrene; ROS: Reactive Oxygen Species.

ROS generation following exposure to 10 µM, 25 µM, 50 µM and 75 µM of BaP concentrations were observed as 160±5.29, 199±19.35, 230±7.23, and 262±5.044% after 2h, 162±7.42, 212±8.76, 234±8.66, and 230±5.77% after 6h, and 140±5.19, 200±2.88, 245±8.66, and 210±2.88% after 12h of exposure and 130±6.35, 170±5.77, 225±8.56, and 140±2.517% after 24h respectively, against unexposed cells, as shown in Figure 6. 50 µM concentration was selected for ROS generation assay in further experimentation as highest ROS induction was observed at this dose.

### 2. MN Assay

**Figure 7:**
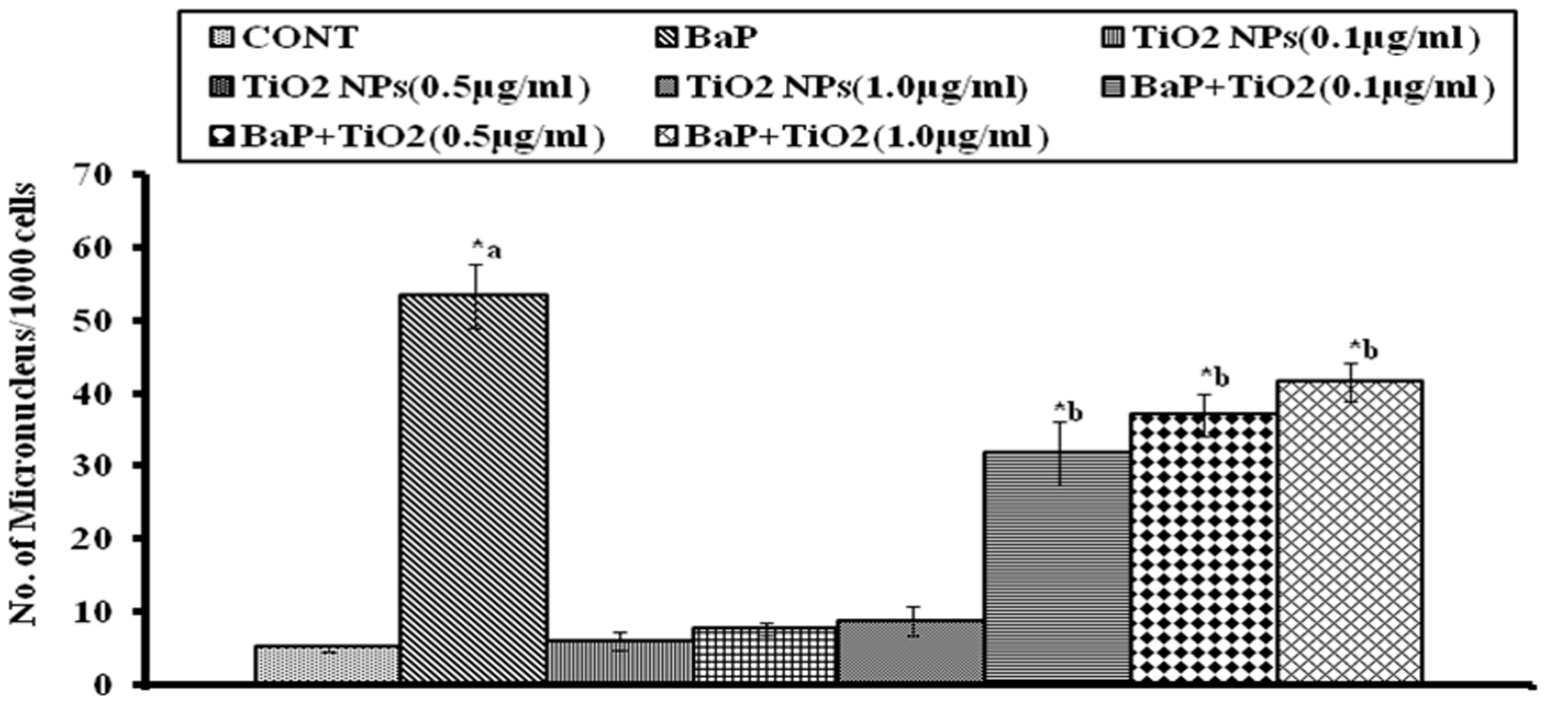
Number of micronucleus/1000 cells after 24 h exposure to 25 µM BaP, to 0.1, 0.5 and 1.0 µg/ml of TiO_2_ NPs, and co-exposure to 0.1, 0.5 and 1.0 µg/ml of TiO_2_ NPs along with 25 µM BaP. *p<0.05 considered as significant. a– as compared to control, b- as compared to BaP treated. BaP: Benzo[alpha]Pyrene; MN: Micronucleus; TiO_2_ NPs: Titanium Dioxide Nanoparticles.

Micronucleus assay was performed to analyze genotoxicity in different experimental sets. Different doses of TiO_2_ NPs (0.1, 0.5 and1.0 µg/ml) induced slightly higher number of MN (6±1.155, 7.66±0.8819 and 8.66±2.028 MN/1000 cells respectively) as compared to unexposed control (5±0.5774 MN/1000 cells). The highest genotoxic dose (25 µM) of BaP induced significantly (p<0.05) high numbers of MN (53.33±4.41 MN/1000 cells). Whereas, co-exposure of BaP (25 µM) and different doses of TiO_2_ NPs (0.1 µg/ml, 0.5 µg/ml, and 1.0 µg/ml) resulted in significant reduction (p<0.05) of MN induction (31.67±4.41, 37±2.887, and 41.67±2.603 MN/1000 cells respectively), as compared to BaP exposed cells as shown in Figure 7.

### 3. Cytotoxicity assay

**Figure 8:**
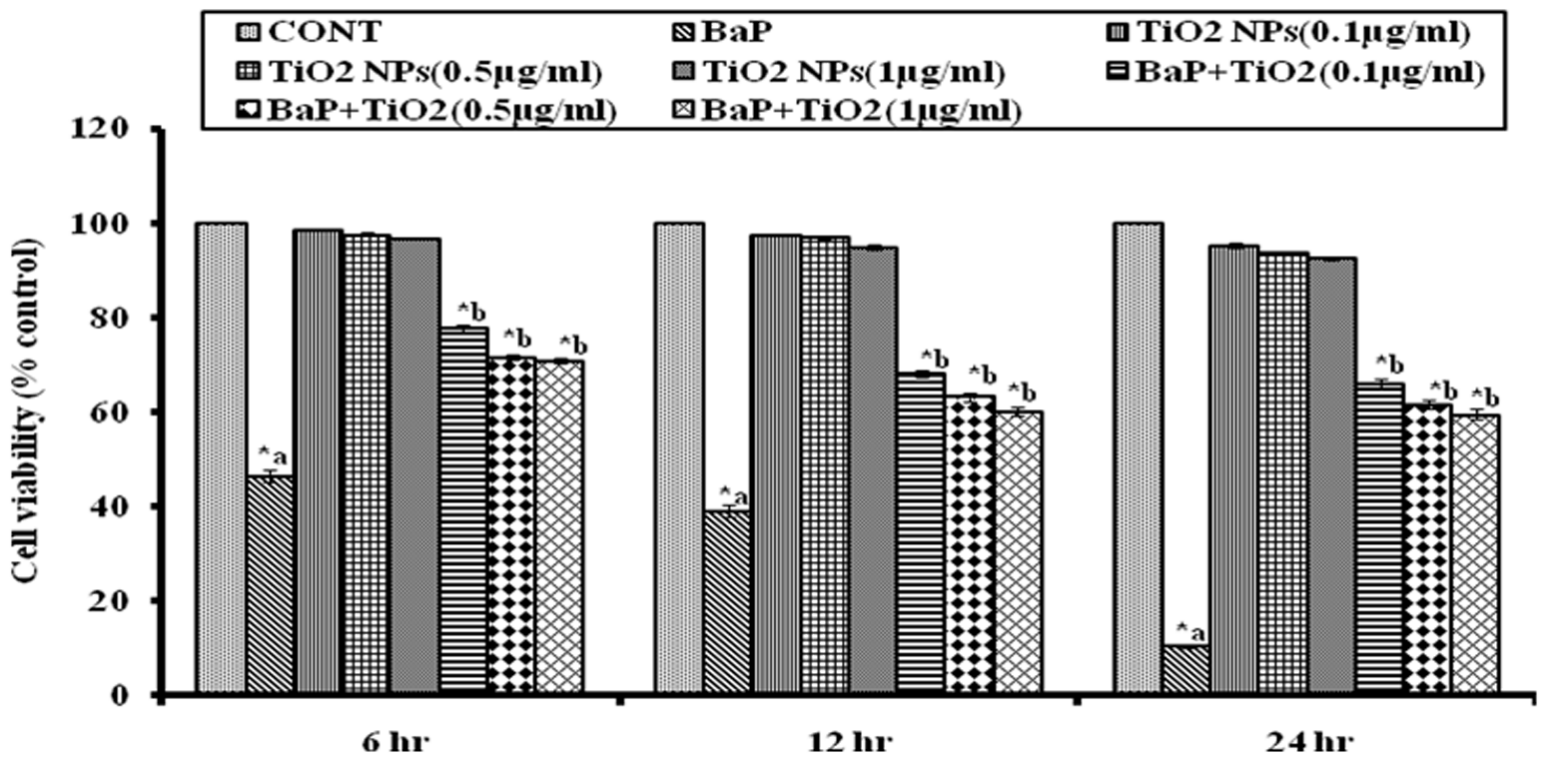
Percentage viability of the cells exposed to 75 µM BaP, to 0.1, 0.5 and 1.0 µg/ml of TiO_2_ NPs and co-exposure to 0.1, 0.5 and 1.0 µg/ml of TiO_2_ NPs along with 75 µM, for 6, 12 & 24 h, as measured by MTT assay. *p<0.05 indicates significance. a– as compared to control, b- as compared to BaP treated. MTT: 3-(4, 5-dimethylthiazol-2-yl)-2, 5-diphenyl tetrazolium bromide; BaP: Benzo[alpha]Pyrene, TiO_2_ NPs: titanium dioxide nanoparticles.

MTT assay was performed to analyze the cytotoxicity in different experimental sets. TiO_2_ NPs exposure resulted in very slight cytotoxicity. The % of viable cell observed after exposure to TiO_2_ NPs (0.1, 0.5 and1.0 µg/ml) was 98.64±0.2022, 97.6±0.3711, and 96.73±0.1802 after 6h, 97.45±0.2461, 96.93±0.3169, and 94.83±0.5185 after 12 h, and 95.3±0.6582, 93.77±0.2963, and 92.47±0.2767 after 24h respectively. But, BaP exposure at selected dose of 75 µM showed significant (p<0.05) reduction in cell viability, which as 46.51±1.147% after 6 h exposure, the effect became more intense after 12h (39.12±1.33%) and 24h (10.41±0.494%) exposure. Whereas, co-exposure of BaP (75 µM) with different doses of TiO_2_ NPs (0.1 µg/ml, 0.5 µg/ml, and 1.0 µg/ml), resulted in significant increase in cell viability (77.7±0.60, 71.72±0.52, and 70.88±0.56% at 6h, 68.16±0.85, 63.31±0.94, and 60.12±0.82% at 12h and 65.85±1.20, 61.73±0.82, and 59.53±1.114% at 24h, respectively) as compared to BaP exposed cells as shown in Figure 8.

### 4. Oxidative Stress

**Figure 9:**
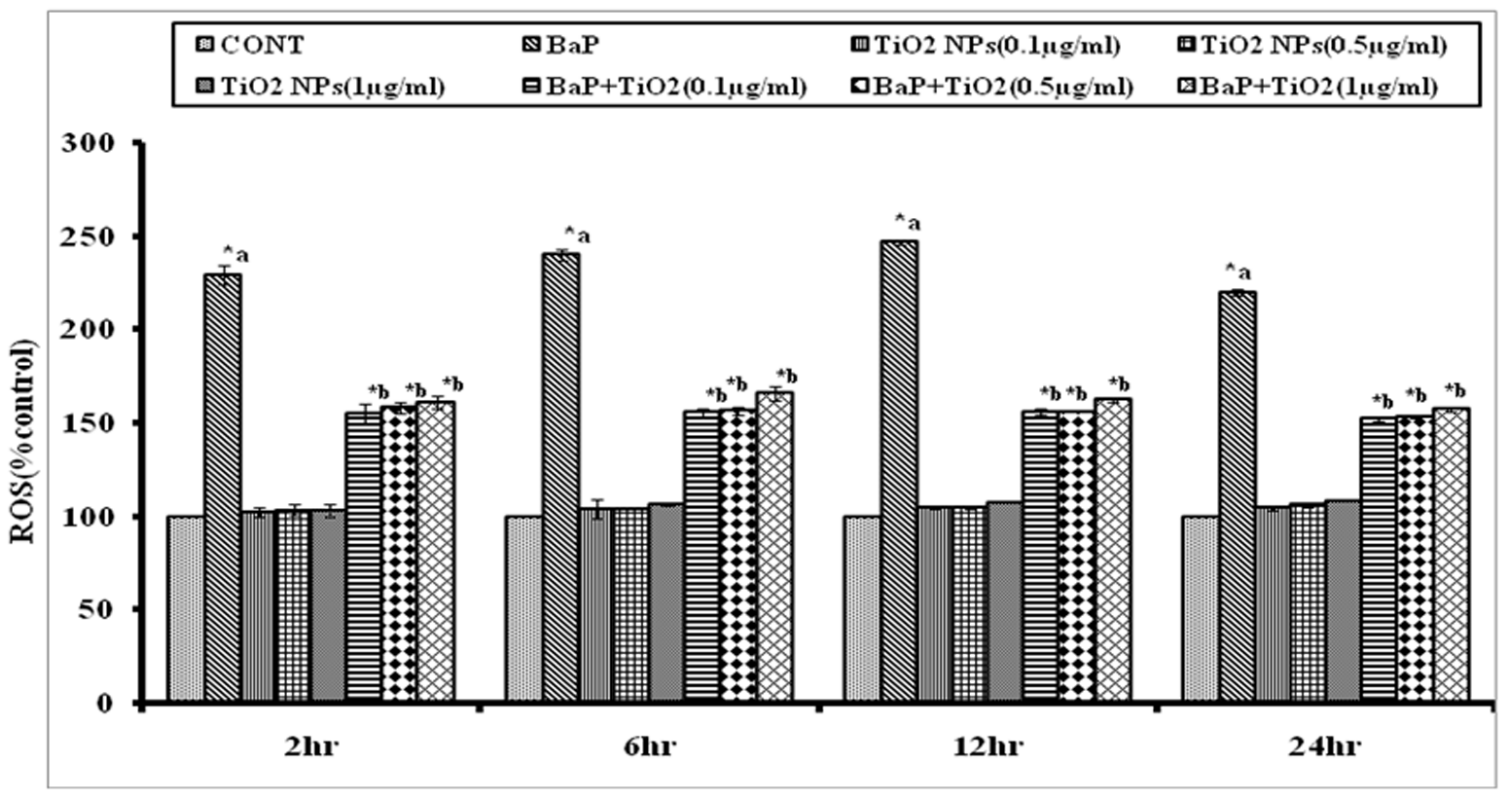
Percentage changes in ROS generation following 6, 12 and 24 h exposure to 50 µM BaP, to 0.1, 0.5 and 1.0 µg/ml of TiO_2_ NPs and co-exposure to 0.1, 0.5 and 1.0 µg/ml of TiO_2_ NPs along with 50 µM BaP, as assessed by DCFH-DA dye. *p<0.05 considered as significant. a– as compared to control, b- as compared to BaP treated. BaP: Benzo[alpha]Pyrene; ROS: Reactive Oxygen Species; TiO_2_ NPs: titanium dioxide nanoparticles.

DCFH-DA staining was performed to analyze ROS generation in different experimental sets. The % of ROS observed after exposure to different doses of TiO_2_ NPs (0.1, 0.5 and1.0 µg/ml) was 102.5±2.201, 103.5±2.871, and 103.3±3.808%, respectively after 2h, 104.5±0.2999, 105±0.4533, and 107.5±0.3143% after 6h, and 104.5±0.2999, 105±0.4533, and 107.5±0.3143% after 12h, 104.6±0.8764, 106.5±1.459 and 108.6±0.2267% after 24h, respectively. The exposure to selected dose (50 µM) of BaP induced significantly (p<0.05) higher ROS (229.2±5.174% after 2h, 240.2±2.976% after 6h, 246.2±1.178% after 12h, and 220.2±1.749% after 24h, respectively). Whereas, the co-exposure of BaP (50 µM) with different doses of TiO_2_ NPs (0.1 µg/ml, 0.5 µg/ml, and 1.0 µg/ml) resulted in significant reduction in ROS generation (154.9±5.71, 158.2±3.17, and 161.4±3.17% after 2h, 155.7±1.78, 156.6±1.79, and 165.9±4.14% after 6h, 156.1±1.76, 156.3±0.24, and 162.6±0.89 after 12h, and 152.5±1.16, 153.6±0.67, and 157.6±1.16% after 24h, respectively) as compared to ROS generation by BaP alone at all time periods, as shown in Figure 9.

### 5. Protein Structure Validation

**Figure 10:**
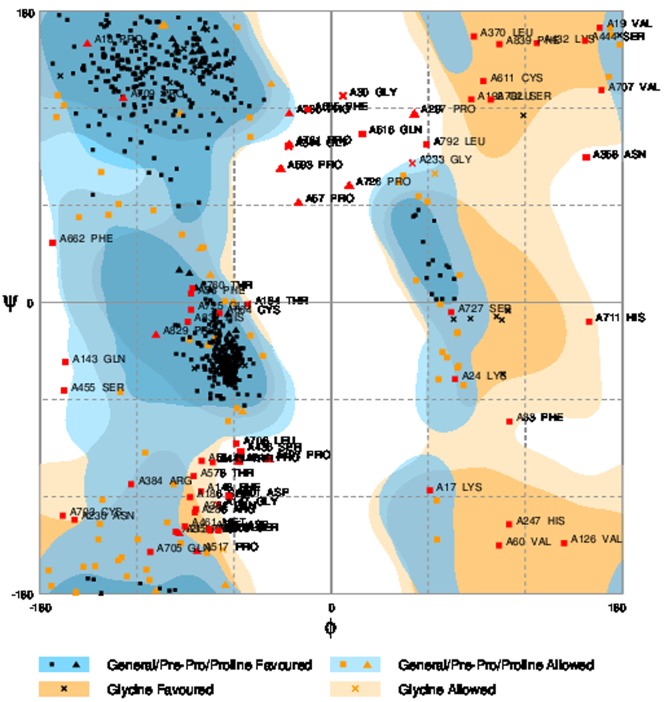
Modeled structures of AHR showing 83.5% of amino acid residues in favored region of Ramachandran plot.

3D structure model of AHR was generated using I-TASSER online server (*ab initio* modeling), as shown in Figure 1. The model was validated using RAMPAGE by Ramachandran plot approach. The torsion angles of the 3D structure of AHR showed 83.5% amino acid residues in the favored regions as shown in Figure 10.

### 6. *In Silico* Docking Studies of TiO_2_ NP and BAP

In the present study, the orientation and binding affinity (in terms of the total docking score and binding residues) of TiO_2_ NP and BaP was explored with AHR.

TiO_2_ NP showed high binding affinity with AHR with a docking score of 12074, as compared to the docking score of BaP with AHR (4600). Docking score of BaP with AHR-TiO_2_ NP complex was 4710.

**Figure 11:**
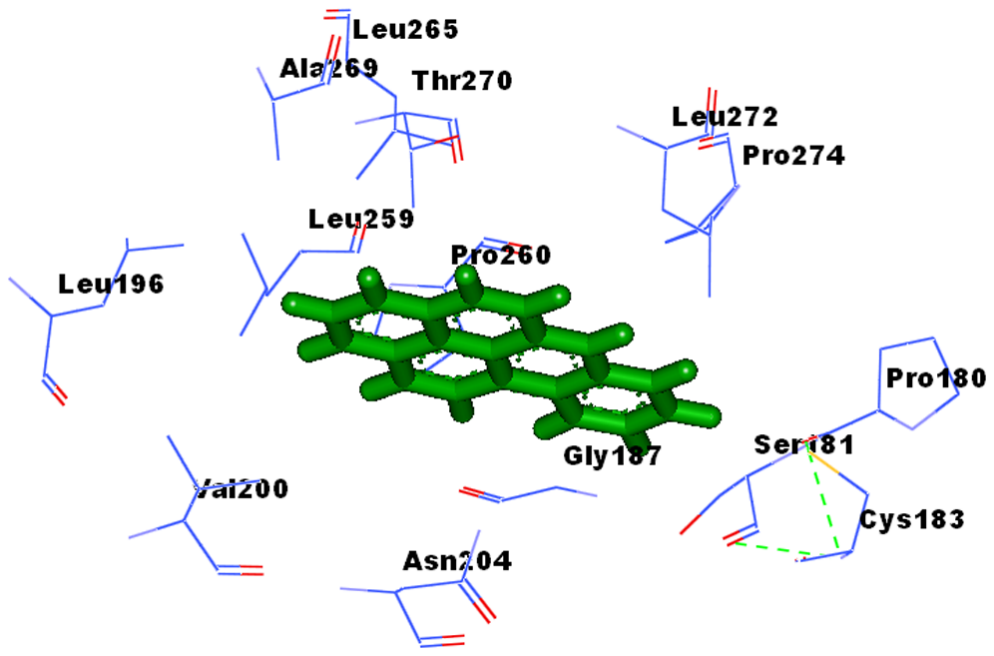
Binding Sites of AHR-BaP complex. (Pro180, Ser181, Cys183, Gly187, Leu196, Val200, Asn204, Leu259, Pro260, Leu265, Ala269, Thr270, Leu272, Pro274).

The chemical nature of binding site residues of AHR within a radius of 4A° with BaP showed hydrophobic interaction with Pro180, Ser181, Cys183, Gly187, Leu196, Val200, Asn204, Leu259, Pro260, Leu265, Ala269, Thr270, Leu272 and Pro274 residues, as shown in Figure 11.

**Figure 12:**
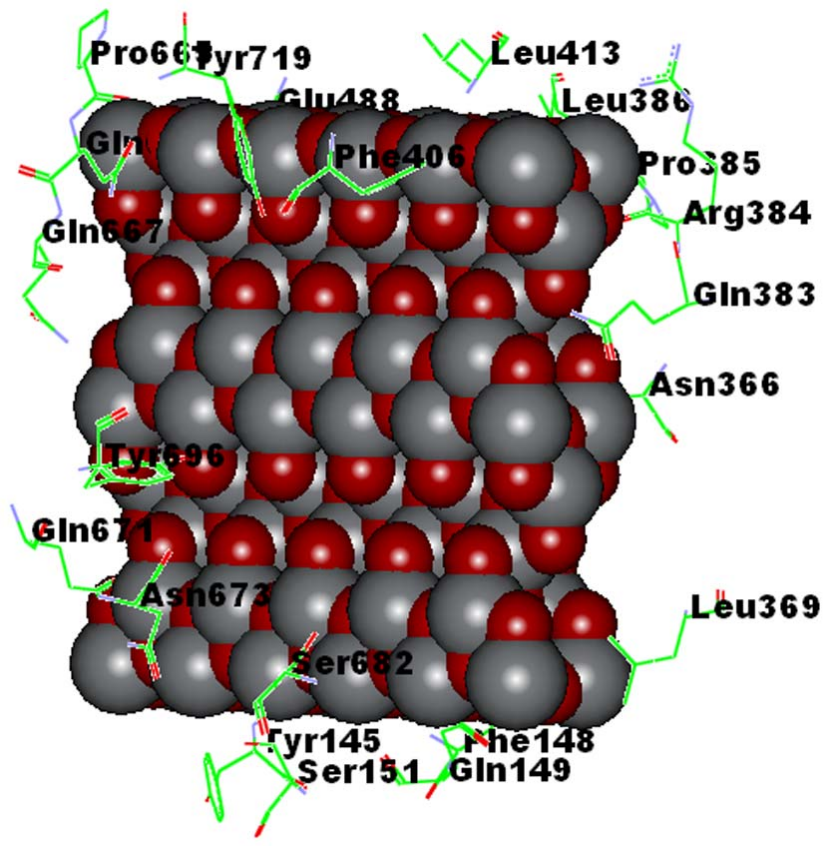
Binding Sites of AHR-TiO_2_ NP complex. (Tyr 145, Ser 151, Phe 148, Leu 369, Asn 673. Asn 366, Agr 384, Pro 385, Leu 413, Try 719, Gln383, Phe 406, Glu 488, Pro 665, Gln 667, Tyr 696, Gln 671, Asp 144, Ser 682, Gln 149).

The chemical nature of binding site residues of AHR within a radius of 4A° with TiO_2_ NP showed hydrogen bond interaction with Gln667-NE2: O98 bond length 3.11 Ǻ, Gln667-N: O38 bond length 2.53 Ǻ, Gln 383- NE2: O75 bond length 1.94 Ǻ, Asp144N: O4 bond length 2.91 Å, Ser 682: OG:O19 bond length 3.26 Å, Gln149 NE2: O19 bond length 2.45 Å, Try 696 CZ-OH: O5 bond length 3.08 Å & O8 bond length 3.08 Å and hydrophobic interaction with Tyr 145, Ser 151, Phe 148, Leu 369, Asn 673. Asn 366, Agr 384, Pro 385, Leu 413, Try 719, Phe 406, Glu 488, Pro 665, Gln 671 & Ser 682, as shown in Figure 12.

**Figure 13:**
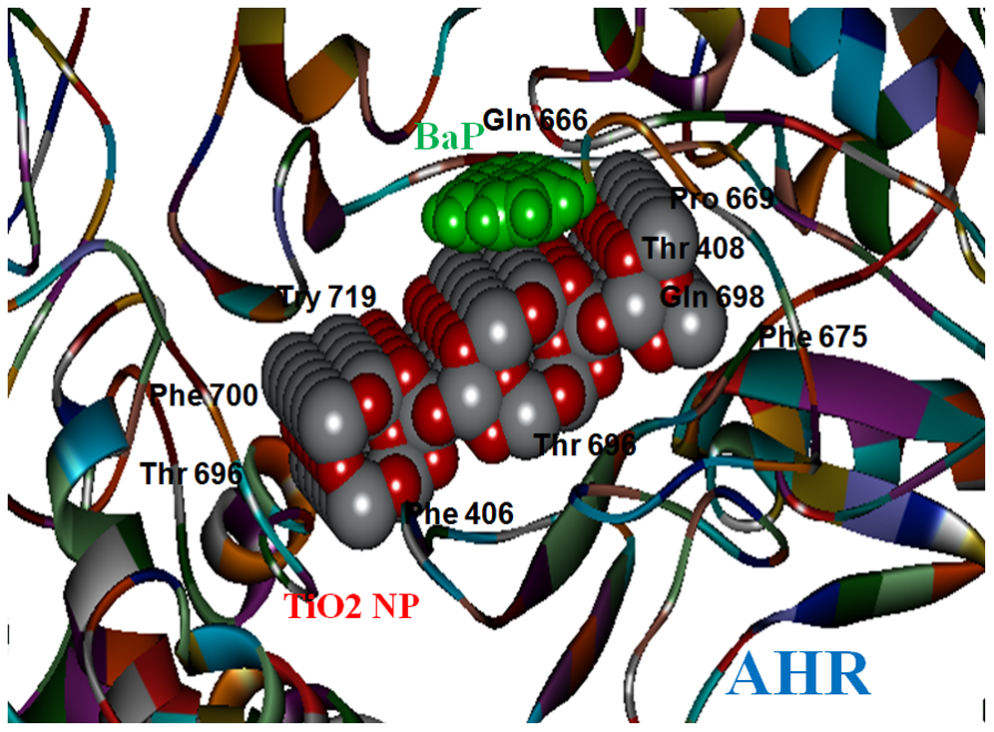
Binding Sites of AHR - TiO_2_ NP complex & BaP. (Gln 666, Try 719, Phe 700, Pro 669, Gln 698, Thr 408, Phe 406, Phe 675, Thr 696).

Whereas, the BaP when docked with AHR-TiO_2_ NP complex was adsorbed at the surface of TiO2 NP, as shown in Figure 13. The chemical nature of binding site residues of AHR-TiO_2_ NP complex within a radius of 4A°, showed the hydrophobic interaction with Gln 666, Try 719, Phe 700, Pro 669, Gln 698, Thr 408, Phe 406, Phe 675, Thr 696.

## Discussion

The present study was designed to explore the probability of protective application of nanoparticles against environmental carcinogen induced toxicity. The doses of BaP, the reference carcinogen in the study, were optimized for MN, MTT and ROS generation assays in A549 cells. The BaP exposure caused significant reduction in cell viability, which was dependent on period of exposure and was highest with 75 µM at 24h (10.41±.494%). This effect could be attributed to enhanced production of ROS as a result of BaP exposure which was found to be highest with 50 µM at 12h (246.9±1.178%). Further, ROS generation might have caused the DNA damage which was highest with 25 µM at 24h (53.33±4.41MN/1000 cells).Which is in accordance with the results of past toxicological studies of BaP [22]. The doses of 25 µM for MN assay, 75 µM for MTT assay and 50 µM for ROS generation assay were selected, as maximum effects were observed at these doses in respective assays.

In order to evaluate the protective effect of nanoparticles, A549 cells were co-exposed to some non-toxic doses of TiO_2_ NPs (0.1, 0.5 and1.0 µg/ml) along with BaP.

At all doses, the TiO_2_ NPs offered protection and raised the viability of A549 cells as compared to viability in only BaP exposed cells as measured by MTT assay after co-exposure. The protective effect was slightly higher with 0.1 µg/ml concentration of TiO_2_ NPs than 0.5 µg/ml and 1.0 µg/ml TiO_2_ NPs.

Similarly, co-exposure of all three doses of TiO_2_ NPs caused significant lowering in ROS production in BaP exposed A549 cells at all time periods. Again, the effect of 0.1 µg/ml concentration of TiO_2_ NPs was marginally higher as compared to 0.5 µg/ml and 1 µg/ml TiO_2_ NPs.

Similar effects were observed in the MN assay also, where co-exposure of all three doses of TiO_2_ NPs caused significant reduction in MN induction by BaP in A549 cells. Again, the effect of 0.1 µg/ml concentration of TiO_2_ NPs was marginally higher as compared to 0.5 µg/ml and 1 µg/ml of TiO_2_ NPs.

All three end points used for study depicted clear cut reduction in the toxicity of BaP, which indicated the protective potential of TiO_2_ NPs in low dose.

**Figure 14:**
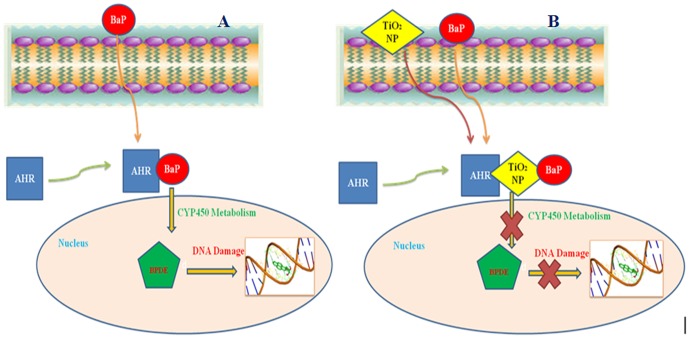
Role of AHR in BaP internalization. (A) Internalization of BaP in to cell through AHR, metabolic conversion to BPDE and interaction of BPDE with DNA. (B) Preferential binding of TiO_2_NP with AHR. TiO_2_ NP bound to AHR blocks the internalization of BaP, preventing its metabolic conversion to BPDE and finally avoiding DNA damage.

*In silico* approach was applied in order to explore the probable mechanism by which TiO_2_ NP provided protection against BaP induced toxicity. Previous studies have established that AHR is responsible for the entry and regulation of the enzymatic metabolism of BaP to Benzo[a]pyrene -7,8-diol-9,10-epoxides (BPDE), a crucial carcinogenic metabolite of BaP, which reacts primarily with the N2 position of guanine residues and to a minor coverage with the N6 position of adenine residues in DNA [8] as shown in Figure 14A. BPDE forms bulky adducts with DNA which blocks DNA synthesis during replication by high fidelity DNA polymerases [3].

The docking study, performed to determine the binding abilities of BaP and TiO_2_ NPs with AHR, revealed that TiO_2_ NP bind with much higher molecular docking score with AHR (12074) as compared with docking score of BaP with AHR (4600). This establishes a strong possibility of preferential binding of TiO_2_ NP over BaP with AHR, incase both TiO_2_ NP and BaP are present together in cell vicinity. Further, to investigate how AHR might respond to BaP when TiO_2_ NP is already bound to AHR, the BaP was docked with AHR-TiO_2_ complex. The BaP was adsorbed strongly at the TiO_2_ NP (score 4710) and not at its original binding site at AHR, as shown in Figure 14B. Previous Studies have also shown a strong adsorption potential of TiO_2_ NP towards PAHs and some other chemical carcinogens present in cigarette [9], [11]. In present case also strong adsorption potential of TiO_2_ NP might have caused the shifting in binding position of BaP from AHR to TiO_2_ bound to AHR.

Once a bulk substance is brought to nano-size, it loses its surface atomic coordinates thereby increasing free surface energy. The stronger binding of the TiO_2_ NP on AHR as well as adsorption of BaP on TiO_2_ surface could be an effect of nanosized TiO_2_ in order to minimize high free surface energy, to accomplish the atomic coordination at the surface and to establish electronic neutrality [23]. Another probable reason for the reduced toxic effect of co-exposure could be the direct adsorption of BaP onto TiO_2_ nanoparticles itself, rendering BaP unavailable to its target molecules, which requires further in depth analysis.

## Conclusion

The present study clearly describes the attenuation of BaP induced toxicity by TiO_2_ nanoparticles in A549 cells, along with the probable mechanism of TiO_2_ NPs protection against BaP. To the best of our knowledge, this is the very first study suggesting future prophylactic application of nanoparticles as guardian against the chemical carcinogens at the molecular/cellular level. We are in process of further investigating whether TiO_2_ NPs are capable of protecting cells against other chemical carcinogens also? And if this process of protection can be further enhanced by modifying the surface chemistry of TiO_2_ NPs. Also, we are exploring the capacities of other nanoparticles like CNT, Fullerene etc. for their potential to provide protection against chemical carcinogens.
